# A Systematic Review of Piezoelectric Materials and Energy Harvesters for Industrial Applications

**DOI:** 10.3390/s21124145

**Published:** 2021-06-16

**Authors:** Abdul Aabid, Md Abdul Raheman, Yasser E. Ibrahim, Asraar Anjum, Meftah Hrairi, Bisma Parveez, Nagma Parveen, Jalal Mohammed Zayan

**Affiliations:** 1 Department of Engineering Management, College of Engineering, Prince Sultan University, P.O. BOX 66833, Riyadh 11586, Saudi Arabia; aaabid@psu.edu.sa (A.A.); ymansour@psu.edu.sa (Y.E.I.); 2 Department of Electrical and Electronics Engineering, NMAM Institute of Technology, Nitte, Karkala Taluk, Karnataka 574110, India; 3 Department of Mechanical Engineering, Faculty of Engineering, International Islamic University Malaysia, P.O. Box 10, Kuala Lumpur 50728, Malaysia; asraar.anjum@gmail.com (A.A.); meftah@iium.edu.my (M.H.); zayan_mohammed@yahoo.co.in (J.M.Z.); 4 Department of Manufacturing and Materials Engineering, Faculty of Engineering, International Islamic University Malaysia, P.O. Box 10, Kuala Lumpur 50728, Malaysia; mirbisma5555@gmail.com; 5 Department of Electrical and Computer Engineering, Faculty of Engineering, International Islamic University Malaysia, P.O. Box 10, Kuala Lumpur 50728, Malaysia; nagmaparveen1192@gmail.com

**Keywords:** piezoelectric energy harvester, piezoelectric materials, smart materials, energy harvesting, modeling

## Abstract

In the last three decades, smart materials have become popular. The piezoelectric materials have shown key characteristics for engineering applications, such as in sensors and actuators for industrial use. Because of their excellent mechanical-to-electrical and vice versa energy conversion properties, piezoelectric materials with high piezoelectric charge and voltage coefficient have been tested in renewable energy applications. The fundamental component of the energy harvester is the piezoelectric material, which, when subjected to mechanical vibrations or applied stress, induces the displaced ions in the material and results in a net electric charge due to the dipole moment of the unit cell. This phenomenon builds an electric potential across the material. In this review article, a detailed study focused on the piezoelectric energy harvesters (PEH’s) is reported. In addition, the fundamental idea about piezoelectric materials, along with their modeling for various applications, are detailed systematically. Then a summary of previous studies based on PEH’s other applications is listed, considering the technical aspects and methodologies. A discussion has been provided as a critical review of current challenges in this field. As a result, this review can provide a guideline for the scholars who want to use PEH’s for their research.

## 1. Introduction

**Figure 1:**
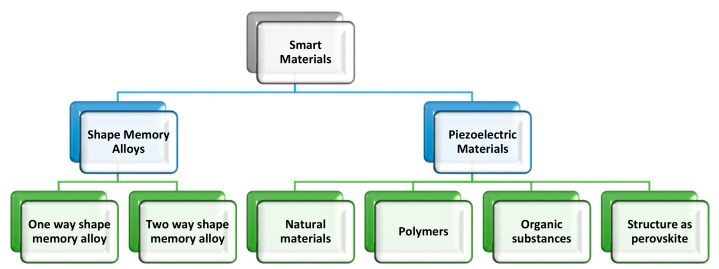
Most common smart materials.

Numerous research efforts have focused on the direction of applications of smart materials in engineering structures. These smart materials possess some attributes, which can be altered desirably under a controlled environment through temperature, stress, and an electric or a magnetic field, which act as external stimuli [1]. The most typical examples of smart materials widely employed in different areas are shape memory alloys and piezoelectric materials, as shown in Figure 1. The latter of the two is characterized by a unique feature referred to as the electromechanical effect. This characteristic is due to the interaction between a given material’s electrical and mechanical properties [2]. For energy harvesting, piezoelectric materials are developing as breakthrough energy harvesters due to their outstanding ability to create electricity from underutilized vibrations of electronics. Today, there is a large choice of piezoelectric materials to select from as a result of the research done on these materials (Figure 1).

This study is focused more on piezoelectric energy harvesters (PEH’s). The term “energy harvesting” refers to the process of transforming other forms of energy in the environment into electrical energy. Power levels of tens of kilowatts may be found in large-scale sources such as car suspension systems, towering structures, and ocean waves. Ambient vibrations or temperature gradients, on the other hand, are incapable of producing power output levels high enough to be considered for power grids. Instead, they can be used to provide clean, long-lasting power to stand-alone electronic sensors or transducer components. This vital role is now done primarily by batteries in current technology examples, but advances in complementary metal–oxide–semiconductor (CMOS) and micro electromechanical systems (MEMS) technologies have permitted the mass manufacture of extremely small sensors and transducers. Current battery technology limits the size, weight, and cost of these devices. Replacement of drained batteries is mostly impractical, limiting the system’s lifespan and long-term viability. As a result, energy harvesting applications are predicted to extend the lifetime of systems or potentially lead to self-sustaining systems by removing the need for batteries entirely, while also providing a huge push for further research in industry and academia [3].

Solar, vibration, radio-frequency, acoustic waves, and temperature gradients are some of the numerous ambient energy sources suitable for energy harvesting applications. Vibrations from the environment can give a high energy density per unit volume of the device. An inertial mechanism can be used to collect vibration energy by coupling the vibration to a proof mass and then dampening the mechanical motion of the mass. Piezoelectric, electromagnetic, and electrostatic energy harvesters are the three basic types of vibration-based energy harvesters. The piezoelectric devices of lower sizes, such as MEMS size devices, benefit from scaling of power with volume since the structures must be manufactured using micromachining processes. The literature has already reported on the power densities that may be achieved using various energy sources, as well as their benefits and drawbacks. For practical applications, piezoelectric vibration energy harvesters are said to have a greater energy density. Another advantage is the capacity to convert reciprocally. PEH’s have been widely researched throughout the last decade. On lesser scales, scaling of power with volume also favors piezoelectric devices.

On the other hand, the piezoelectric devices used as mainstream materials in smart actuator systems, such as miniature ultrasonic motors, precision positioners, and adaptive mechanical dampers, are reported in this study. The piezoelectric and related ceramic actuators mainly concentrate on improving the materials of the actuator, the shape, design, structure, and their applications. Piezoelectric actuators have developed a new field of electronic and structural ceramics. Another promising application area is the suppression of vibrations in military vehicles and space structures using piezoelectric actuators. Recently, temperature-controlled solid-state displacement transducers (shape memory alloy) have been tested, but they are inferior to piezoelectric ceramics [4]. Yoichi et al. [5] in their study, has stated that the piezoelectric actuator extends beyond industrial machinery. They concluded that it could be used in portable electronic devices such as digital cameras and mobile phones. Studies are progressing rapidly towards many applications where piezoelectric materials are used alongside nanomaterials in various fields of application. They are intended to use a piezoelectric material in sizes of nanometers to be used as actuators which can be the first multilayering, multi-tasking material that can lead to technological advances in vast applications such as industrial equipment and digital home appliances.

Due to its versatile advantage and the vast applications of piezoelectric materials, a systematic literature review has been carried out in this study based explicitly on energy harvesting in engineering structures. Energy harvesting using lead zirconate titanate (PZT) as a piezoelectric material has become very common in the last five years. In this review, Section 2 discusses piezoelectric materials, constitutive equations, and the types of their configuration. Section 3 lists the theoretical modeling of the piezoelectric transducer. Section 4 states the energy harvesting studies for different objects with fundamental ideas and their relations. Section 5 elucidates the discussion with limitations and challenges in this field and relates to the present review contents. Finally, a conclusion has been constructed based on the current review work.

## 2. Piezoelectric Materials

Piezoelectric materials are simple, low cost, lightweight, and easy-to-control smart material for structural actuation application. It is known for its adaptability in a vast range of applications in different structures; piezoelectric materials can easily be molded into various forms such as patches, thin films, cylinders, and fibers. The piezoelectric material which is commonly used in automotive and aerospace engineering nowadays are: Lead Zirconate Titanate (PZT), Lead Titanate (LT), Sodium Potassium Niobate (SPN), Lead Magnesium Niobate (PMN), and Leadmetaniobate (LMN). Of the five listed types of piezoelectric material above, Lead Zirconate Titanate (PZT) is one of the most frequently studied ferroelectric materials due to its vast array of applications as a pyroelectric material which is widely used for the repair of the structure [6]. From the literature, we observed that PZT is being used for the repair of cracks, shape control, vibration control, and structural health monitoring [7].

Many piezoelectric materials have been created over the last century, but perovskite lead zirconate titanate which is a polycrystalline monolithic piezoelectric ceramic known as PZT is the most popular one and is often doped with niobium or lanthanum to form soft and hard piezoelectric materials. While PZT is the most prevalent material, it contains lead, necessitating a substantial and continuous research effort to find other formulations [8,9,10,11]. Recently, Gao et al. [12] developed a PNN PZT(0.55Pb(Ni_1/3_Nb_2/3_)O_3_–0.135PbZrO_3_–0.315PbTiO_3_) ceramic with a remarkably high coupling coefficient much higher than conventional PZT ceramics. While piezoelectric ceramics are inexpensive and offer good coupling, they are brittle and dense. PZT thin films have been created to leverage on the small scale to produce flexibility, as well as the use of grain texturing and epitaxial thin films on substrates to increase coupling, due to the expanding application of piezoelectric ceramics in MEMS [13,14,15].

Although there are ways for generating high-quality piezoelectric films, there is still a lot of work being done on optimizing material deposition for 3D transducers, creating lower-temperature production processes and alternative substrate materials, and enhancing electrode texturing [16]. In comparison to dense piezoelectric, porous piezoelectric materials have greater hydrostatic piezoelectric strain and voltage coefficients, making them a good choice for hydrostatic sensors like active and passive sound navigation and range (SONAR) [17,18,19]. Additionally, piezoelectric polymers have been created to produce compliant piezoelectric materials, including polyvinylidene fluoride (PVDF). While piezoelectric polymers are light and flexible, their coupling is significantly weaker than that of ceramics. The use of a near field electrospinning process to prepare PVDF material was suggested to improve the coupling coefficient of these materials by an order of magnitude [20]. Harstad et al. [21] devised a new strategy to increase PVDF polymer coupling by raising the β phase percentage in the material composition. The improvement is obtained by employing a phase-inversion approach to synthesize Gd_5_Si_4_-PVDF nanocomposite. Recently, simple, low-cost, and repeatable processes involving solvent casting, screen printing, replica molding, and electrospray/electrospinning methods were used to create P(VDF-TrFE) microstructures with various topologies and morphologies [22,23,24].

Solvent casting and screen-printing processes were used to create porous and dense films with varied solvent evaporation temperatures. Prefabricated molds acquired using typical microfluidic processing technologies were used to produce patterned P(VDF-TrFE) microstructures via replica molding. Microstructures ranging from semi-spheres to fibers were created using electrospray/electrospinning processes, with the copolymer content varied. The various microstructures obtained through the various techniques used do not significantly affect the copolymer phase, degradation, and melting temperature, degree of crystallinity, dielectric constant, and piezoelectric coefficient of the samples, according to the physicochemical and electrical characterization. As a result, these structures show great promise in a variety of biotechnological applications, including biomedical, energy storage, sensors and actuators, and filtration, to name a few [22]. Apart from PZT and PVDF, the bulk of piezoelectric energy harvesting transducer materials may be divided into five categories: single-crystal piezoelectric, lead-free piezoelectric, high-temperature piezoelectric, piezoelectric nanocomposites, and piezoelectric foams which have been discussed in detail in [25].

### 2.1. Piezoelectric Constitutive Equation

The constitutive equations of the PZT property assume that the total strain in the transducer is the sum of mechanical strain induced by the mechanical stress and the controllable actuation strain caused by the applied electric voltage [26].

The direct piezoelectric effect is the capability of piezoelectric materials to create an electric field under the influence of mechanical stress. This property of the piezoelectric materials is utilized to generate electrical energy when strain is applied. The reciprocal of the direct piezoelectric effect is the inverse piezoelectric effect in which mechanical strain is developed in response to the electric field [27]. These effects are strongly dependent on the crystal orientation concerning the strain or electric field. In many cases, a robust piezoelectric effect is observed in one orientation. Poling axis, i.e., the direction of polarization, is parallel to the z-axis. The standard piezoelectric notation used is that the x, y, and z axes correspond to subscripts 1, 2, and 3. Hooke’s law is used to describe the piezoelectric material’s electrical behavior and is represented as [28],

(1) D=εE

where D is the electric polarization, ε is the permittivity, and E is the applied electric field strength. In order to describe a system, Hooke’s Law states that:

(2) S=sT

where S is the strain, s is the compliance, and T is the stress. Equations (1) and (2) are combined to form the following relations:

(3) $$\left\{\begin{array}{c} \left\{D\right\}=\left[d^{t}\right]\left\{T\right\}+\left[\varepsilon^{t}\right]\left\{E\right\}\\ \left\{S\right\}=\left[s^{E}\right]\left\{T\right\}+\left[d\right]\left\{E\right\} \end{array}\right.$$

where $\left[d\right]$ is the direct piezoelectric coefficient matrix, $\left[d^{t}\right]$ is the matrix that describes the converse piezoelectric effect, *E* is the electric field vector, *T* is the stress vector, and *t* determines the transposition matrix.

A more straightforward approach for describing the direct and converse piezoelectric effect is represented as [29],

(4) $$\left\{\begin{array}{c} D=dT+\varepsilon E\\ S=sT+dE \end{array}\right.$$

**Figure 2:**
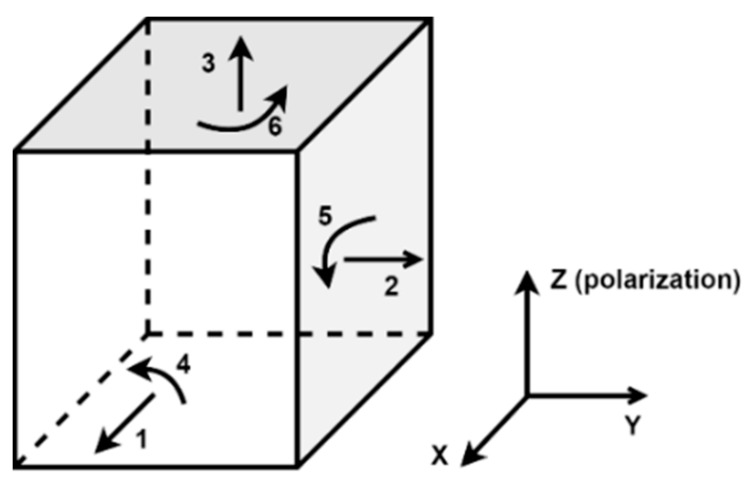
Structure of piezoelectric material.

The constitutive equation represents the direct piezoelectric effect,

(5) D=dT+εE

where D is the electric polarization (C/m^2^), d is a piezoelectric coefficient matrix, T is the stress vector (N/m^2^), ε is the electrical permittivity matrix (F/m), and E is the electric field vector (V/m). By convention, the polarization direction of the piezoelectric material is depicted by direction 3, as shown in Figure 2.

Mechanical strain and an electric field’s predicted directions in a piezoelectric material are also designated using the specified indices. For example, if a piezoelectric material operates in d_31_ mode, it indicates that the output is sensed across the material on axis 3 due to stress on axis 1. Here, the electrical output is proportional to the coefficient d_31_; hence it is named d_31_ mode. Equation (5) implies that the charge produced in the piezoelectric material is proportional to the stress applied. As a result, the PEH’s are built to optimize stress under a specific mechanical load. The transducer’s mechanical-electrical conversion efficiency $E_{\%}$ determines the performance of PEH’s [30], which can be calculated as [31],

(6) $$E_{\%}=\frac{P_{out}}{P_{in}}\times 100$$

where *P_out_* is the electrical output power, defined as,

(7) $$P_{out}=v_{p}i_{p}$$

and *P_in_* is the mechanical input power, defined as,

(8) $$P_{in}=Fv$$

where $v_{p}$ is the overall voltage generated between the transducer’s electrodes, $i_{p}$ is the current supplied by the piezoelectric transducer, F is the external mechanical force, and v is the speed of the moving object.

### 2.2. Types of Piezoelectric Material Configurations

**Figure 3:**
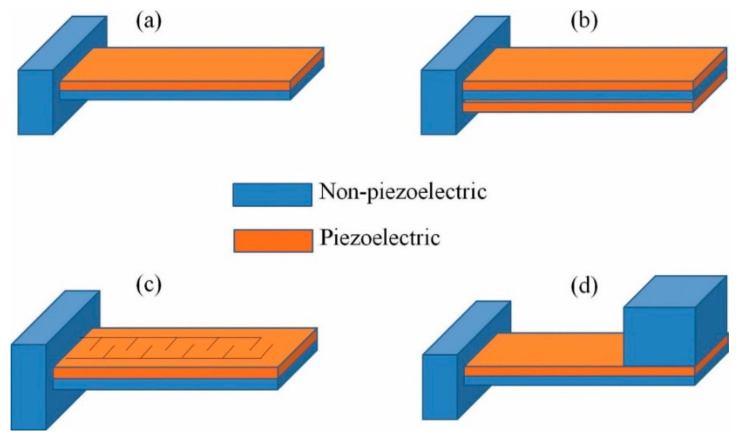
Cantilever beam transducer (**a**) unimorph; (**b**) bimorph; (**c**) cantilever with interdigitated electrodes; (**d**) cantilever with proof mass at its free end. Reprinted from [34], Copyright 2021, with the permission of AIP Publishing.

**Figure 4:**
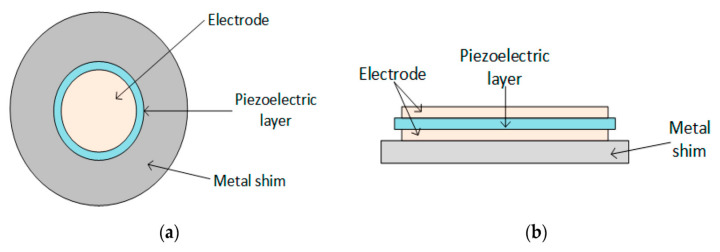
Circular diaphragm transducer: (**a**) Front view; (**b**) Side view [32]. Reprinted under the Creative Commons (CC) License (CC BY 4.0).

**Figure 5:**
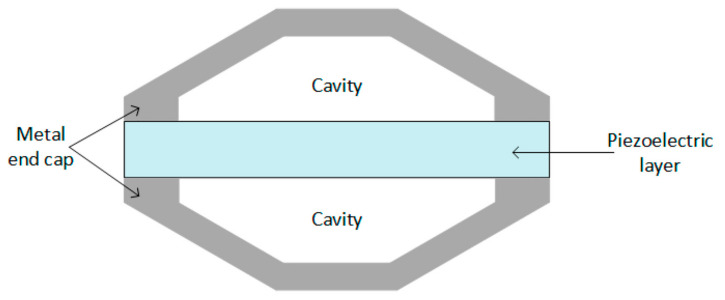
Cymbal type transducer [32]. Reprinted under the Creative Commons (CC) License (CC BY 4.0).

**Figure 6:**
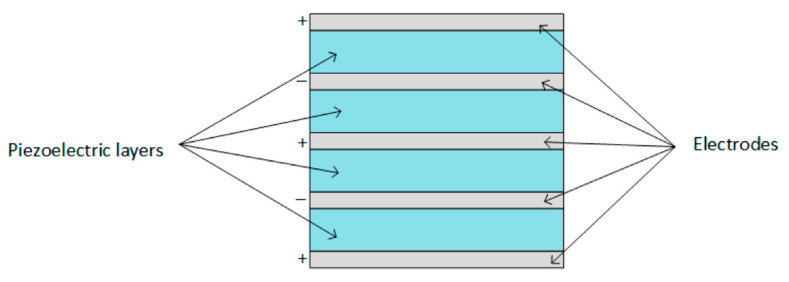
Stack type transducer [32]. Reprinted under the Creative Commons (CC) License (CC BY 4.0).

Piezoelectric materials transducers are available in the following configurations [32]: Cantilever beam (Figure 3), circular diaphragm (Figure 4), cymbal type (Figure 5), and stacked type (Figure 6).

Cantilever beam configuration is the most common for PEH’s with either one or more layers termed as unimorph and bimorph (Figure 3a,b), respectively, due to the high mechanical strain during vibration and the relatively simple construction. The fundamental flexural modes of a cantilever have a much lower resonance frequency than the piezoelectric element’s other vibration modes. Because of these advantages, the majority of PEH’s use this configuration. Figure 3a shows a cantilever constructed with a thin layer of piezoelectric ceramics, bonded to a non-piezoelectric layer (usually a metal acting as a conductor for the produced charge), and one end fixed to use the structure’s flexural mode. Since only one active layer (the piezoelectric layer) is included in the structure, it is referred to as a “unimorph.” Figure 3b shows two thin layers of piezoelectric ceramic bonded to the same metal sheet to create a cantilever that maximizes the unit’s power output. Since two active layers are used, this configuration is referred to as a “bimorph” structure. The bimorph configuration doubles the energy capacity of PEH without significantly increasing the unit volume, and hence bimorph piezoelectric cantilevers are more widely used in PEH studies [33].

Piezoelectric cantilevers operate in the “31 mode”, where “3” denotes the polarization direction of the piezoelectric layer and “1” denotes the direction of the stress, which is predominantly in the cantilever’s planar direction, and the “31 mode” uses the $d_{31}$ piezoelectric charge constant. In the 31 modes, stress is not applied along the polar axis of piezoelectric material; hence $d_{31}$ is always smaller than $d_{33}$ for a given material. Therefore, an interdigitated electrode design can use a piezoelectric layer in the “$d_{33}$” mode for higher energy production (Figure 3c). In an interdigitated electrode design, alternate layers of narrow positive and negative electrodes are placed in an array on the surface of the piezoelectric sheet. During poling treatment of the sheet, these interdigitated electrodes direct the electric field to apply laterally within the sheet such that the sheet is polarized in the lateral direction instead of the conventional vertical direction. Therefore, when the sheet is subjected to bending, the stress direction is parallel to the poled direction of the piezoelectric, enabling the utilization of the primary piezoelectric charge constant, $d_{33}$. A simply supported cantilever beam’s resonance frequency may be computed as [34,35,36,37].

(9) $$f_{r}=\frac{v_{n}^{2}}{2\pi }\frac{1}{L^{2}}\sqrt{\frac{EI}{mw}}$$

where E is Young’s modulus, I is the moment of inertia, L is the length, w is the width of the cantilever, m is the mass per unit length of the cantilever beam, and $v_{n}=1.875$ is the mass per unit length of the cantilever beam the eigenvalue for the fundamental vibration mode.

A seismic mass is usually attached to the free end of the cantilever to adjust the resonant frequency to the available environment frequency, which is usually below 100 Hz (Figure 3d). Equation (9) can be approximated to include the seismic mass as [37],

(10) $$f_{r}=\frac{{v^{\prime }}_{n}^{2}}{2\pi }\frac{1}{L^{2}}\sqrt{\frac{K}{m_{e}+∆m}}$$

where ${v^{\prime }}_{n}^{2}=v_{n}^{2}\sqrt{0.236/3}$, $m_{e}=0.236\;mwL$ is the effective mass of the cantilever, ∆m is the seismic mass, and K is the effective spring constant of the cantilever.

A cantilever’s energy output depends mainly upon seismic mass and the volume of the piezoelectric material subjected to mechanical stress [38,39]. During bending of a cantilever, induced stress is concentrated near the clamped end of the cantilever [39]. The strain is maximum at the clamped end and decreases in magnitude at locations further away from the clamp. Therefore, the non-stressed portion of the piezoelectric layer does not contribute to power generation. A tapered or triangular cantilever form has been shown to produce a consistent strain level along the length of the cantilever in both theoretical and experimental research [40,41,42,43]. Hence, a tapered shape piezoelectric cantilevers have often been used to minimize the size and weight of the cantilever.

Figure 4 shows the circular diaphragm structure, which consists of a thin disk-shaped piezoelectric layer attached to a metal shim fixed on the edges of the clamping ring. A proof mass is attached at the core of the diaphragm to improve the power output and the performance under low-frequency operation [33]. Figure 5 shows cymbal transducers used in applications that have high impact forces. It typically consists of a piezoelectric ceramic disc and a metal (steel) end cap on each side. Steel has higher yield strength than brass and aluminum, achieving high load capability in the transducer [34,44]. Multiple piezoelectric layers are stacked over each other to form stack piezoelectric transducers. Layers are stacked such that the poling direction of the layers aligns with the applied force (Figure 6). These transducers are used in applications which demand higher pressure [33].

The comparison of each configuration with advantages and disadvantages has been illustrated in Table 1. It shows that each type of configuration has its edge, which is suitable for many applications. At the same time, however, it should be noted that it might be disadvantageous in other applications. Hence, the limitations should be studied before application.

## 3. Piezoelectric Material Modelling

**Figure 7:**
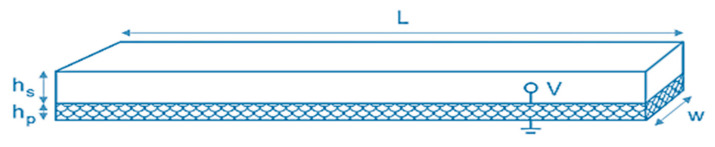
Piezoelectric mono-morph plate.

Inoue et al. [45] studied the double-coated layers of Pb (Zr, Ti) O_3_ (PZT) thin films and reported the influence of increased thickness on the piezoelectric bimorph structure. The thick piezoelectric films function effectively in microelectromechanical systems (MEMS) applications, allowing high-voltage application and large generative force. Enhancement and electrode structures promote the development of the biomorphic thin-film PZT/PZT structure. Hudec [46] studied the mono morph (also called uni-morph) piezoelectric plate used to analyze the piezoelectric mono-morph, their geometry, and an overview of their underlying physics. The piezoelectric mono-morph is composed of two layers, the first one is called the substrate layer made of pure silicon-coated by a thin layer of gold, and the coating is for X-ray reflective optics. The second layer is a piezoelectric actuator, microfiber composites M-8557-P2. The dimensions are shown in Figure 7. For the analysis of the cantilevered mono-morph, including the free length of l mm, which is shorter than the total length L, due to the rigidity, clamping is required. The parameters used to simplify this: L = 100 mm, I = 95.5 mm, hs = 0.7 mm, hp = 0.3 mm, and w = 63 mm.

A simply supported beam with voltage and the parameters are derived. The materials’ beam and piezoelectric layer properties were studied [47]. The derivation was done both mathematically and numerically. The effect of various parameters on the voltage needed was investigated. By considering a beam of height H with a piezoelectric layer of thickness δ attached at the bottom of the beam over the entire length of beam L, Lee et al. [48] derived certain relations. The charge that is produced as a sensor by the piezoelectric layer can be written as,

(11) $$Q=-e_{31}\overset{L}{\underset{0}{\int }}\left(\frac{H+\delta }{2}\right)y^{\prime \prime }\mathrm{dx}$$

where $e_{31}$ is the piezoelectric constant, the voltage output of the sensor, vs. is given by [49]

(12) $$V_{s}=\frac{Q}{C_{v}}=\frac{e_{31}\left(H+\delta \right)}{2C_{v}}\overset{L}{\underset{0}{\int }}y^{\prime \prime }\mathrm{dx}$$

where $C_{v}$ is the electrical capacitance.

When the piezoelectric layer is used as a collocated sensor and actuator, the voltage applied to the piezoelectric layer, V_a_, can be written as.

(13) $$v_{a}={\mathrm{gv}}_{s}=-s\;\frac{e_{31}\left(H+\delta \right)}{2}\overset{L}{\underset{0}{\int }}y^{\prime \prime }\mathrm{dx}$$

where g is the control gain factor and s = g/Cv. The axial stress that the applied voltage induces along the piezoelectric layer can be expressed as [50].

(14) $$\sigma_{x}=e_{31}\frac{v_{a}}{\delta }=-s\frac{e_{31}^{2}}{2\delta }\left(H+\delta \right)\overset{L}{\underset{0}{\int }}y^{\prime \prime }\mathrm{dx}$$

This affects the bending moment on the beam given by

(15) $$M_{a}=\sigma_{x}\delta \left(\frac{H+\delta }{2}\right)=-\frac{{\mathrm{se}}_{31}^{2}}{2}{\left(H+\delta \right)}^{2}\overset{L}{\underset{0}{\int }}y^{\prime \prime }\mathrm{dx}=-{\left.{\mathrm{Gy}}^{\prime }\right|}_{x=0}^{x=L}$$

where,

(16) $$G=\frac{{\mathrm{Se}}_{31}^{2}}{4}{\left(H+\delta \right)}^{2}$$

If a small patch is a piezoelectric layer, M_a_ is a local moment, proportional to the slope shift (i.e., curvature) caused by the actuator.

### 3.1. Piezoelectric Coulomb’s Friction Modeling

For the study of multi-domain BEM, a Coulomb’s friction modeling with the formulation of the boundary integral method was introduced to in-plane electrostatic and generalized plane strain elasticity assumptions for a 2D piezoelectric domain, with boundary ∂Ω, and two-faced in the $x_{1}x_{2}$ plane [51]. For the intention of succinctness, the governing equations of the problem are not presented in the current study; nevertheless, this can be found in Alaimo et al. [51,52]. By applying the conditions of compatibility and consistency on all sub-sections boundaries (interface), the global system of equations about the total gathered structure is then taken:

(17) $$\delta_{\partial \Omega_{ij}}^{\left(i\right)}=\delta_{\partial \Omega_{ij}}^{\left(j\right)}=\Delta \delta^{ij}\;i=,\ldots ,N-1$$

(18) $$P_{\partial \Omega_{ij}}^{\left(i\right)}=-P_{\partial \Omega_{ij}}^{\left(j\right)}\;j=i+1,\ldots ,N$$

**Figure 8:**
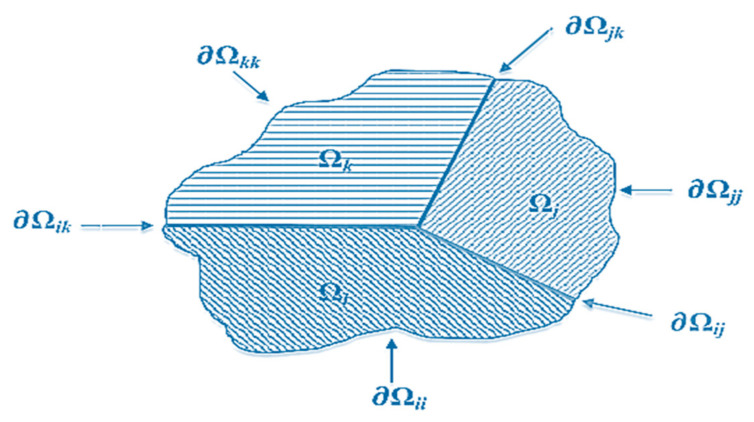
Multi-domain assembling [51]. Reprinted under the Creative Commons (CC) License (CC BY 3.0).

The subscript, $\partial {Ω}_{ij}$, implies the numbers related to the boundary (interface) nodes between the sub-sections of *i*^th^ and *j*^th^; see Figure 8.

**Figure 9:**
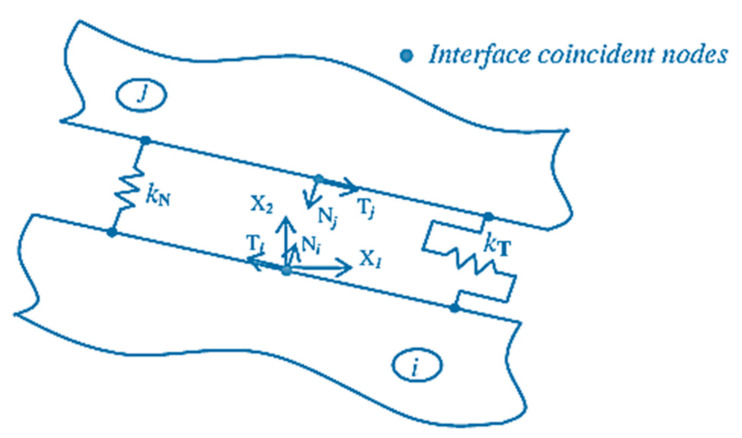
Interface local reference system and spring model [51]. Reprinted under the Creative Commons (CC) License (CC BY 3.0).

More precisely, by considering the local reference structure of Figure 9, by stating the normal and tangential components of the interface displacement (mechanical) jumps, $∆\delta_{N}^{\left(ij\right)}$ and $∆\delta_{T}^{\left(ij\right)}$, the spring model is implemented as a purpose of the normal and tangential components of the nodal mechanical traction components:

(19) $$∆\delta_{N}^{\left(ij\right)}=k_{N}P_{N}^{\left(i\right)}\;with\;∆\delta_{N}^{\left(ij\right)}=\delta_{N}^{\left(i\right)}-\delta_{N}^{\left(j\right)}$$

(20) $$∆\delta_{T}^{\left(ij\right)}=k_{T}P_{T}^{\left(i\right)}\;with\;∆\delta_{T}^{\left(ij\right)}=\delta_{T}^{\left(i\right)}-\delta_{T}^{\left(j\right)}$$

### 3.2. Piezoelectric Sensor Modeling

The sensor modeling is a part of piezoelectric material’s governing equations which expresses the sensing purposes with lower electric fields. To measure or monitor any device, either intact or damaged, this expression is employed. Therefore, to study the sensor modeling, a sample study from the previous investigation has been redrawn. It shows the sensor measurements at distinct positions at which the modal states are determined. The sensor in the form of the strain gauge is positioned at both sides of the third buckling model location with four samples: x = L/3 and 2L/3 for the supported beam and x = L/5 and 3L/5 for the supported the cantilevered beam. For both cases, a total of four samples of strain gauge sensors were utilized. During the installation of sensor modeling, a third mode with their multiples is unmeasurable, whereas, in the case of the 1st, 2nd, 3rd, and 5th models of beam buckling, the modal control is realized. Hence, it has been recognized that with the use of these strain gauge measurements, the performance of the beam can be determined. For a two-beam model (simply supported and cantilevered) sensor output vectors can be derived as follows,

(21) $$w_{n}\left(x,\;t\right)={\;\varphi }_{n}\left(x\right)q_{n}\left(t\right)=\sqrt{\frac{2}{L}}\;\sin\left(\frac{n\pi x}{L}\right)q_{n}\left(t\right)$$

The above Equation (21) represents the standardized un-forced dynamics modal for the simply supported beam and is determined from the theory of dynamic beam dynamic [53]. Next, the equation bending moment ($M_{b}$) with a strain gauge positioned is expressed as,

(22) $$M_{b}=-E_{b}{\;I}_{\mathrm{eq}}w^{\prime \prime }\left(x,\;t\right)=\sqrt{\frac{2}{L}}E_{b}{\;I}_{\mathrm{eq}}\frac{\pi^{2}}{L^{2}}\overset{n=\infty }{\underset{n=1}{\sum }}n^{2}q_{n}\;\sin\;\left(\frac{n\pi x}{L}\right)$$

where $E_{b}$ is the modulus of elasticity for a beam, $I_{\mathrm{eq}}$ is a moment of inertial (equivalent) beam. For the beam materials, a composite piezo-beam segment considered for investigation, and the distance of x is measured from the left-side end of the beam. The location of the strain gage is at the single side of the beam, then the resulting strain is determined by

(23) $$\varepsilon \;=\pm \frac{M_{b}\left[\frac{t_{b}}{2}+{\;t}_{p}\right]}{E_{b}{\;I}_{\mathrm{eq}}}-\frac{P}{A_{\mathrm{eq}}E_{b}}$$

where $A_{\mathrm{eq}}$ is a beam material-based equivalent area, the symbol of the initial term of Equation (23) differs on which side the bond is on the strain gauge. Then the output is independent of the load P of the variance of strain gage for the simply supported beam and is given by,

(24) $$\mathrm{vs}\;=2{\varepsilon k}_{g}=2\sqrt{\frac{2}{L}}k_{g}\frac{\left[\frac{t_{b}}{2}+{\;t}_{p}\right]\pi^{2}}{L^{2}}\overset{n=\infty }{\underset{n=1}{\sum }}n^{2}q_{n}\;\sin\;\left(\frac{n\pi x}{L}\right)$$

where the constant of the strain gauge is $k_{g}$ and the n^th^-order modal co-ordinate is $q_{n}$. The output vector of the device is,

(25) $$V_{0}=\left\{\begin{array}{c} V_{01}\\ V_{02} \end{array}\right\}=\left\{\begin{array}{c} v_{s}(x=\frac{L}{3})\\ v_{s}(x=\frac{2L}{3}) \end{array}\right\}$$

Analogously, for the cantilevered beam, the output of a strain gauge can be extracted as

(26) $$\mathrm{vs}\;=\frac{1}{2}\sqrt{\frac{2}{3L}}k_{g}\frac{\left[\frac{t_{b}}{2}+{\;t}_{p}\right]\pi^{2}}{L^{2}}\overset{n=\infty }{\underset{n=1,3,5,\ldots }{\sum }}n^{2}q_{n}\;\cos\;\left(\frac{n\pi x}{2L}\right)$$

and the output vector in the system is,

(27) $$V_{0}=\left\{\begin{array}{c} V_{01}\\ V_{02} \end{array}\right\}=\left\{\begin{array}{c} v_{s}(x=\frac{L}{5})\\ v_{s}(x=\frac{3L}{5}) \end{array}\right\}$$

### 3.3. Piezoelectric Finite Element Modeling

It is important to know the piezoelectric material equations for FE modeling. Since the PZT transducer is a type of anisotropic material, it has fundamental derivative terms used to evaluate the sensing and actuating purposes during FE analysis. For such an example, this study considered an Euler-Bernoulli beam dynamic buckling equation subjected to a lateral bending load with a steadily rising axial compressive load (p), and it is given by,

(28) $$\rho A\frac{\partial^{2}y}{\partial t^{2}}+\mathrm{EI}\frac{d^{4}w}{{\mathrm{dx}}^{4}}+p\frac{d^{2}w}{{\mathrm{dx}}^{2}}=p\left(x,t\right)$$

where $w\left(x,\;t\right)$ is the beam transverse displacement, ρ is the density of mass per length, and A is the beam cross-sectional area, EI is the beam rigidity, $p\left(x,\;t\right)$ is the externally applied lateral load. Following the theory of the Euler-Bernoulli beam, a transverse direction cubic displacement field is presented as,

(29) $$w\left(x,\;t\right)=\;H\left(x\right)*d\left(t\right)=\overset{4}{\underset{i=1}{\sum }}H_{i}\left(x\right)d_{i}^{e}\left(t\right)$$

It is now possible to express the consequent FE Equations as,

(30) $$\left[M^{e}\right]\left\{{\ddot{d}}^{e}\right\}+\left[K^{e}\right]\left\{d^{e}\right\}-p\left[K_{G}^{e}\right]\left\{d^{e}\right\}=\left\{f^{e}\right\}$$

where $H_{i}\left(x\right)$ is the Hermitian cubic form functions; the reliable mass matrix, the bending stiffness matrix, the force vector element, and the geometric stiffness matrix are as follows, respectively [54],

$$\left[M^{e}\right]=\rho \frac{Al}{420}\left[\begin{array}{cccc} 156 & 22l & 54 & -13l\\ 22l & 4l^{2} & 13l & -3l^{2}\\ 54 & 13l & 156 & -22l\\ -33l & -3l^{2} & -22l & 4l^{2} \end{array}\right]$$

$$\left\{f^{e}\right\}=\overset{l}{\underset{0}{\int }}p(x,t)\left\{\begin{array}{c} H_{1}\\ H_{2}\\ H_{3}\\ H_{4} \end{array}\right\}dx\;\left[K^{e}\right]=\frac{EI}{l^{3}}\left[\begin{array}{cccc} 12 & 6l & -12 & 6l\\ 6l & 4l^{2} & -6l & 2l^{2}\\ -12 & -6l & 12 & -6l\\ 6l & 2l^{2} & -6l & 4l^{2} \end{array}\right]\;\left[K_{G}^{e}\right]=\frac{1}{30l}\left[\begin{array}{cccc} 36 & 3l & -36 & 3l\\ 3l & 4l^{2} & -3l & -l^{2}\\ -36 & -3l & 36 & -3l\\ 3l & -l^{2} & -3l & 4l^{2} \end{array}\right]$$

$$\left[K^{e}\right]=\frac{EI}{l^{3}}\left[\begin{array}{cccc} 12 & 6l & -12 & 6l\\ 6l & 4l^{2} & -6l & 2l^{2}\\ -12 & -6l & 12 & -6l\\ 6l & 2l^{2} & -6l & 4l^{2} \end{array}\right]$$

$$\left[K_{G}^{e}\right]=\frac{1}{30l}\left[\begin{array}{cccc} 36 & 3l & -36 & 3l\\ 3l & 4l^{2} & -3l & -l^{2}\\ -36 & -3l & 36 & -3l\\ 3l & -l^{2} & -3l & 4l^{2} \end{array}\right]$$

The global matrix equation forms after the assembly of element matrices and vectors, as given by,

(31) $$\left[M\right]\left\{\ddot{d}\right\}+\left[K\right]\left\{d\right\}-\;P\left[K_{G}\right]\left\{d\right\}=\left\{F\right\}$$

The above Equation (31) is well known as the beam coupled FE governing equation for piezoelectric actuator modeling. Such an example was found in the case of a vibration analysis of structures by FE modeling of piezoelectric patches [55], active vibration control (AVC) [56,57,58,59,60,61,62,63], and dynamic structural control can also be found in many cases [64,65,66,67,68,69].

## 4. Piezoelectric Energy Harvesters

This section discussed the direct piezoelectric effect and some recent trends and techniques in piezoelectric energy harvesters (PEH’s).

Typically, PEH’s have two parts [70], the first part is a transducer that generates electrical energy, and the second part is a circuit that converts and rectifies the generated circuit. Hence, a PEH’s performance depends on both the transducer as well as the circuit. The PEH’s, in general, consist of three phases, mechanical to mechanical energy conversion, mechanical to electrical conversion, and electrical to electrical energy conversion [30,71]. Various piezoelectric materials can be used in PEH’s. Table 2 presents peak power generated by piezoelectric materials: PVDF, piezoelectric ceramic, piezoelectric fiber, PMN-piezoelectric single crystal, and PMN-PT single crystal. The piezoelectric transducer is found in various configurations such as cantilever beam, cymbal type circular diaphragm, and stack type [32]. Table 2 provides a comparison of these configurations.

Anton and Sodano presented a comprehensive review of power harvesting techniques using piezoelectric materials from 2003 to 2006 [77]. The authors compared various piezoelectric materials with their advantages, disadvantages, and power harvesting capabilities. The authors also investigated various tuning schemes, their advantages and disadvantages. Further, they discussed various PEH applications such as synchronous switch harvesting on an inductor (SSHI), a PEH in implantable and wearable power supplies, a PEH using ambient fluid flows, and a PEH in microelectromechanical systems. Toprak and Tigil presented a comprehensive review of macro and mesoscale PEH’s, MEMS-scale PEH’s, and nanoscale PEH’s [3]. The authors provided a comparison of different resonant modes of cantilever-type PEH’s. They discussed various issues of PEH’s, such as bandwidth, CMOS compatibility, and biocompatibility of the PEH’s, and proposed multiple methods to overcome the problems.

Safaei et al. [25] presented a comprehensive review of energy harvesting using piezoelectric materials from 2008 to 2018. The authors discussed various piezoelectric materials, their properties, various piezoelectric energy harvesting methods and designs, energy conditioning circuits, their energy generation capability, and the applications of PEH’s. The authors comprehensibly discussed various PEH’s such as piezoelectric beam harvesters, piezoelectric transducer configurations, nonlinear and broadband piezoelectric harvester designs, piezoelectric-MEMS energy harvesting designs, energy harvesters based on meta-material and meta-structure, windmill-style PEH’s, flutter-style PEH’s, piezoelectric devices for liquid flow energy harvesting, wearable PEH’s, implantable PEH’s, PEH’s from animals, PEH designed for infrastructure, PEH designs for energy harvesting from vehicles, multifunctional piezoelectric energy harvesting systems, multi-source energy harvesters, and alternative PEH’s.

**Figure 10:**
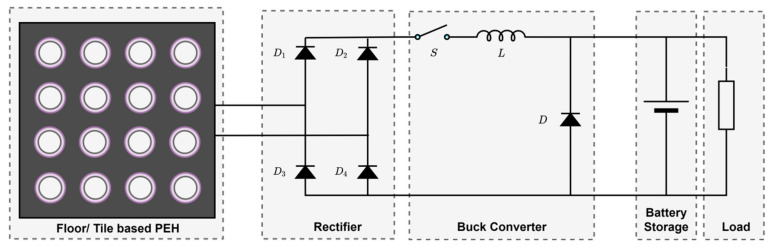
Floor/tile-based PEH.

A typical floor or tile embedded PEH system [78] is shown in Figure 10, in which stress is exerted on the piezoelectric transducers attached to the tiles when a person walks on them. Kamboj et al. [79] proposed a footstep energy harvesting system using piezoelectric transducers. The generated energy was converted to the 220 V alternating voltage. Elhalwagy et al. [80] presented the feasibility analysis of PEH embedded in the floor tiles in the interiors of a building. The authors concluded that electrical power could be generated from a few microwatts to a few watts per step using floor tile-based PEH’s, depending upon the piezoelectric technology, pedestrian frequency, and area. Table 3 presents various piezoelectric technologies and generated energy.

**Figure 11:**
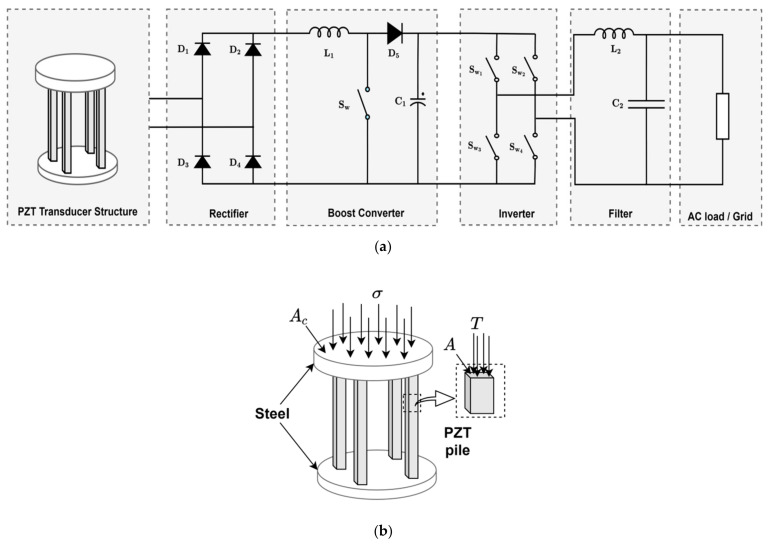
Energy harvesting using piezoelectric transducers (**a**) vehicular moment system. (**b**) transducer structure.

**Figure 12:**
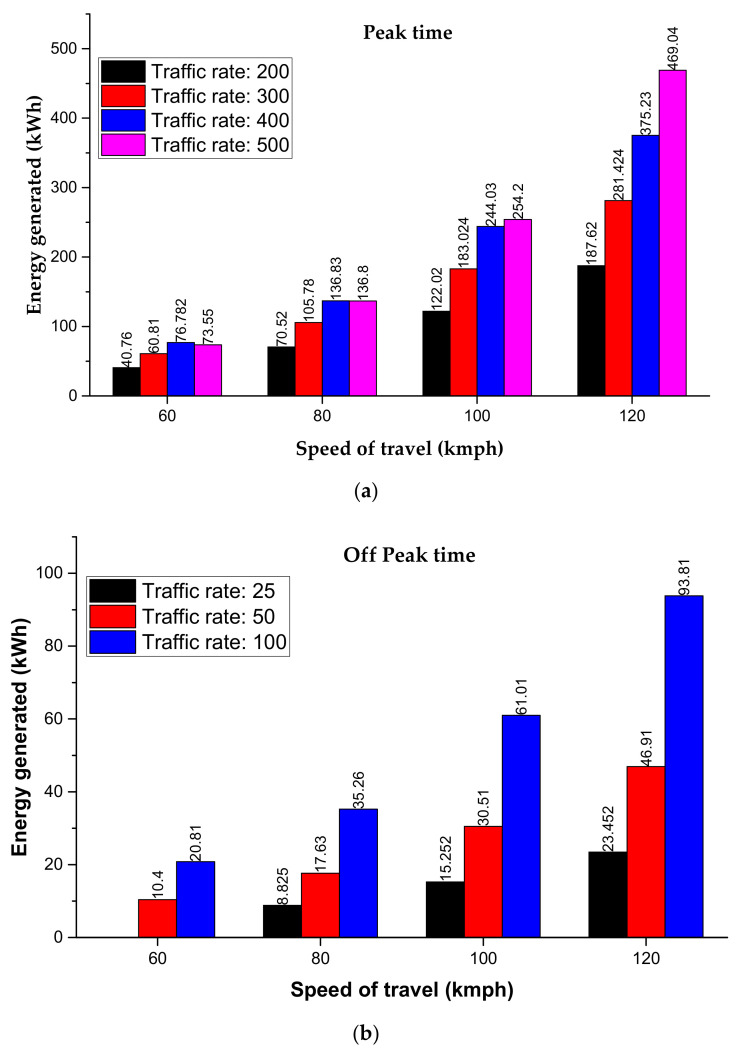
Power generation using vehicular movement in Dubai traffic at different traffic rates/hr (**a**) peak time duration (**b**) off-peak time duration.

Figure 11 shows an energy harvester system using a piezoelectric transducer via vehicular movement [81,82]. To extract the energy efficiently from vehicular movement using PEH’s placed under the asphalt pavement environment, Zhang et al. [83] recommended using a PZT transducer that consists of 8–16 PZT piles for a pavement area ($A_{c}$) of 0.04 m^2^ arranged between steel plates as shown in Figure 11a. The stress T experienced by the piles of PZT shown in Figure 11b is given by Equation (30):

(32) $$T=\frac{\sigma A_{c}}{nA}$$

where σ, $A_{c}$, n, and A are vertical stress on the pavement, effective load area, number of PZT piles in a harvester, and area of PZT, respectively. Najini et al. [84] performed the techno-economic analysis of piezoelectric energy generation from vehicle traffic under different traffic conditions in Dubai, as shown in Figure 12.

Shoe-mounted piezoelectric energy harvesting systems have been developed for various low power applications such as medical sensing [85]. The energy harvester powered the electronic devices. The harvester generated energy per step in a few µJ. The authors of [85] demonstrated that wearable sensor electronic devices could be adequately powered through a piezoelectric energy-harvester. Vinolo et al. [86] presented a novel idea of generating electrical energy from oceanic kinetic energy through buoy motion. The authors proposed a new technique where the energy generated by the low-cost disk piezoelectric transducers does not depend on their excitement frequency.

Ba et al. [87] developed a novel printing paper-like PEH’s by combining natural cellulose nanofibrils. Natural cellulose nanofibrils and barium titanate (BTO) nanoparticles were used to create a piezoelectric composite film. The authors then proposed a roll-coating and screen-printing process based on the printing fabrication. The method used was simple, cost-effective, and gave a high throughput. A hybridized piezoelectric film was made using a polarization treatment process by a charging arrangement of electric dipole moments, causing an external electric field of 3.6 MV/m. The authors observed that the BTO concentration affects the magnitude of the voltage output generated, and 25% was found as the optimal concentration.

**Figure 13:**
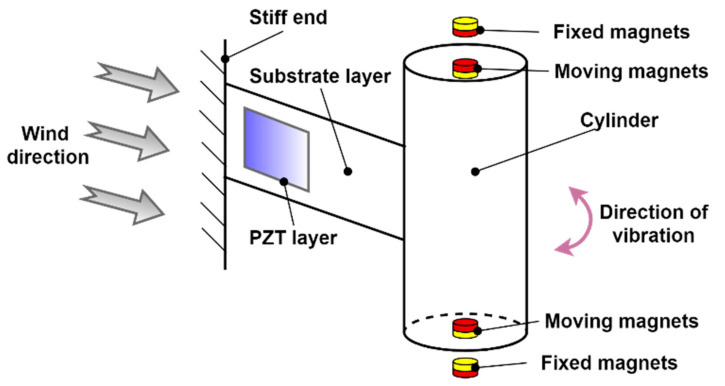
Sketch of magnetic-coupling piezoelectric energy harvester (MCPEH) system [90]. Reprinted under the Creative Commons (CC) License (CC BY 4.0).

Kim et al. [88] analyzed the feasibility of existing coastal structures for power generation using piezoelectric transducers. The authors also examined the recent trends in oceanic wave generators. They conducted the hydrographic analysis using a 2D wave flume in a hydraulic model experiment. From the results, they confirmed that the higher wave with a more extended period of wave produces maximum voltages. Yi et al. [89] proposed thinned piezoelectric films on flexible polymer substrate-based vertically integrated double buckled-bridge PEH. The proposed strategy provided stable high-energy output per chip area for the energy harvester. The outcome was improved due to the low stiffness clamping, which in turn benefits the reversal of the buckled bridge to induced large deformation. Hou et al. [90] proposed, modeled, and analyzed a magnet-induced monostable nonlinear vortex-induced vibration (VIV) PEH as shown in Figure 13. The authors investigated the effect of load resistance, cylindrical structure, piezoelectric beam, and wind velocity on the vibration response and the performance of the energy harvester. The authors found that with an increase in cylindrical diameter and the length of piezoelectric and a decrease in cylindrical mass, the resonant wind velocity increases. The output power of the magnetic-coupling piezoelectric energy harvester (MCPEH) and the steady-state of the system was influenced by the nonlinear magnetic force.

In the aerospace industry, one of the primary considerations in minimizing the malfunctioning of an aircraft is its health control. This requires many types of sensors, which need to be custom-built based on the airplane type. An autonomous energy source is needed; hence, many energy harvesting techniques were developed. The usage of piezoelectric materials in aircraft sensors, health control, and energy harvesters was discussed by Elahi et al. [91]. They provide a detailed understanding of how piezoelectric materials can be used in the aerospace industry at different levels. An energy storage concept containing a piezoelectric patch on a vertical cantilever beam with a tip mass was proposed. They explored the random noise excitation amplitude where the harvester could not hold the high amplitude solution and could jump to the low amplitude solution at some point [92].

**Figure 14:**
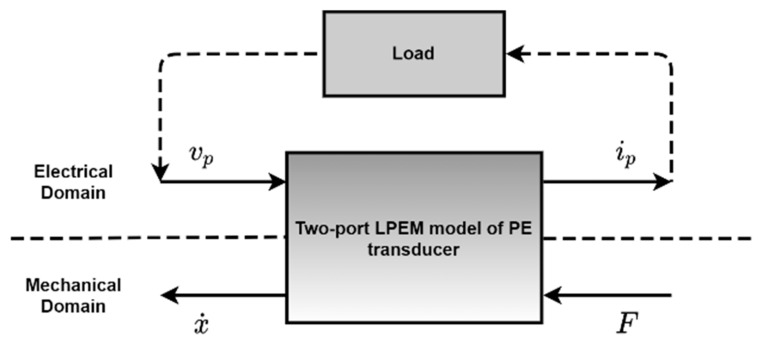
A two-port network model for the PE transducer. Reprinted from [93], Copyright 2021, with permission from Elsevier.

Tavares and Ruderman [93] performed a feasibility analysis of the energy harvesting system utilizing piezoelectric transducers. The authors formulated and implemented a two-port model, and from experimental data, model parameters were identified. The model was based on the lumped parameter electromechanical (LPEM) model shown in Figure 14. The authors were able to observe the expected hysteric transient behavior of the model. The derived model accurately predicted the electrical and mechanical output variables; the author suggested its further use in the design and analysis of a piezoelectric- based energy harvesting system.

## 5. Discussion and Critical Reviews

A comprehensive review of thin piezoelectric films based on MEMS energy harvesters was conducted by Todaro et al. [94], highlighting the strategies and approaches for the harvester. The authors suggested that for harvesting energy from environmental vibrations, these harvesters should be compact and cost-effective. An extensive review of nanostructured polymer-based piezoelectric and triboelectric materials was studied. These materials were lightweight, flexible, biocompatible, lead-free, and cost-effective [95]. The effect of various parameters such as nanoconfinement, surface polarization, growth parameters, self-poling, and device assembly on PEH performance was highlighted. Piezoelectric materials are widely used in multiple applications with distinctly different aerospace, automotive, and medical characteristics. This review category can assist researchers in gaining a global understanding of piezoelectric material research areas, particularly in the early stages of research.

The development of wireless technologies, wearable devices, implants, and internet of things concepts resulted in a need for self-powered systems. The capability of batteries is limited and is not always available in hard-to-reach areas. Thus, the solution is to use self-powered systems. One of the ways to power such systems is to use piezoelectric actuators to harvest mechanical energy. However, the piezoelectric actuator-based energy harvesters have several disadvantages: low voltage generation, low power generation, and the need for additional circuitry for rectification, extraction of maximum power, and regulation of maximum voltage. Hence there is a vast scope for research in selecting the piezoelectric material, configuration of the transducer and optimally design a piezoelectric energy harvester for a specific application. The existing literature provides a clear picture of the progressive production of piezoelectric actuators in energy harvesting. Energy harvesting is one of the areas of application in which these actuators have been progressively used, which is clear from the considerable number of research conducted on them. Most of the previous works related to dynamic reactions of piezoelectric coupled systems with open-circuit electrical boundary conditions were performed using FE simulations.

In recent studies, Abuzaid et al. [2,96,97,98,99,100] used piezoelectric material to control the damage propagation in aerospace structures. The ideas were developed to produce compression/tension stress around the damaged area along with the width of the piezoelectric actuator. The authors stated that increasing the piezoelectric voltage will result in a decrease of fracture parameters such as stress intensity factor [101] and stress concentration factor [102], but the supplied voltage increase was only up to 150 V in their studies [100,103,104] due to limited voltage. The piezoelectric actuators have the advantage to control/repair the damaged aerospace structures, but it has a limitation of the voltage. Hence, in such cases, the PEH’s can be utilized to increase the voltage range to decrease the fracture parameters for the betterment of structures.

### 5.1. Limitations in Piezoelectric Energy Harvesters

**Figure 15:**
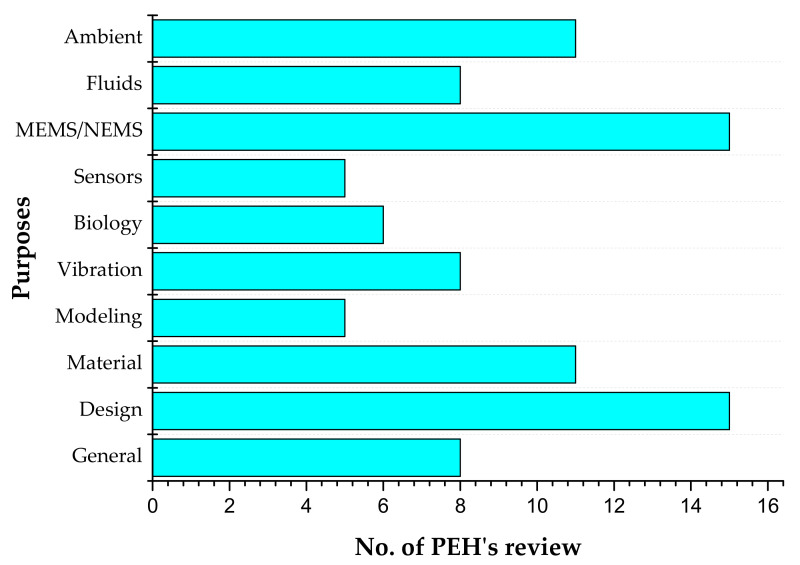
Number of review papers published on different topics related to PEH’s.

This paper has reviewed various PEH’s for different applications. Figure 15 shows the number of review papers published on different topics related to PEH’s. The topic and period of the publications are general: 2005–2019; design: 2005–2020; material: 2009–2019; modeling: 2008–2017; vibration: 2004–2015; biology: 2011–2019; sensors: 2007–2016; MEMS/NEMS: 2006–2019; fluids: 2013–2020; and ambient: 2014–2020.

The future PEH’s have to overcome the current limitations. Table 4 illustrates the recent developments in PEH’s, techniques adopted, and the limits of such PEH’s. Some of the significant drawbacks of the present PEH’s are that they generate lesser power at low voltages than other energy harvesting techniques. The resonant frequency of the few PEH’s is relatively low, and hence frequency tuning and frequency-up techniques are required. The mechanical constraints of the piezoelectrical materials and PEH structure limit the energy output of the PEH’s, and the selection of material also plays a crucial role in the performance of the PEH’s. The overall efficiency of the PEH’s not only depends on the piezoelectric transducer but also on the additional circuitry. Hence the optimal design of circuitry is also required to get the higher efficiency of PEH’s.

### 5.2. Challenges in Piezoelectric Energy Harvesters

Based on the previous researchers’ methods either experimental, simulation, or analytical for implementing the PEH’s in practical applications, they have faced some challenges which are illustrated below:

1. The majority of vibrational energy harvesters are intended to operate in resonance mode, with a narrow half-power bandwidth [3].
2. One of the problems with conventional energy harvesting systems is that they only have one energy conversion mechanism, therefore, their performance is highly dependent on ambient energy levels, which can have a significant influence on the system’s performance [25].
3. Due to the large deformation on the piezoelectric cantilever beam, the voltage, and power generated show nonlinear behavior. The gap between the cover plate and stopper also influences the energy output. An increase in the gap steadily increases the energy output, but a larger gap can make walking on the tiles too uncomfortable. Hence, the gap width should be optimized between high energy generation and comfortable walking [109].
4. The buoy is an aerologic device that is restricted to heave motion only. The vertical motion of the buoy causes the vertical oscillations of the mass. This vertical motion of the buoy is due to the displacement motion of particles caused by the waves. These oscillations deflect the piezoelectric beams. The energy generated by the harvester depends on the deflection of the piezoelectric beams, which in turn depends on the ocean wave force and amplitude, which is related to the wave height [114].
5. The scholar shows that the piezoelectric wave-energy converter generates electricity only when the wave height is greater than 15 mm [116].
6. The unpredictable and low rotational frequency of a tire, space constraints for harvester deployment, and low average output power of a harvester are the main challenges of piezoelectric energy harvesting from a tire [120].
7. The significant problems for piezoelectric energy harvesters, according to Rezaei et al. [121], are the low input frequencies of mechanical energy sources and the difficulty of getting piezoelectric harvesters to respond to them efficiently, as well as the performance limit of piezoelectric materials.
8. The development of an appropriate packaging solution for MEMS-based piezoelectric energy harvesters has a number of challenges, including [122]: Keeping yields from being lost during dicing and device separation; the energy harvesters should be packed in a way that allows for unrestricted flexibility; connection of the energy harvester to the appropriate electrical outlets to get electricity; the inclusion of appropriate integration possibilities in larger electronic systems; protection of the devices from the external environment.

These challenges can be focused on in future studies, as well as finding their solutions in industrial applications. Additionally, one of the main important points that should be noted regarding PEH’s is the type of ductile material that cannot be used in the high-stress area which will result in breaks of the PEH’s specimen [7,101].

## 6. Conclusions

Based on the present investigations, the following conclusions have been made,

- Piezoelectric energy harvesters use the direct piezoelectric effect to generate electrical energy under the influence of mechanical stress. Some of the recent trends and techniques in self-powered devices have been utilizing them.
- The performance of the piezoelectric energy harvesters depends on numerous factors such as the materials selected, type of configuration, mechanical constraints of materials and structure, and the design of the additional circuitry.
- A recent trend in piezoelectric energy harvesters has been studied, and the focus of research, techniques used, and their limitations have been tabulated.

In summary, guidelines for scientists using piezoelectric energy harvesters with various structural devices are presented in this study. The critical literature of piezoelectric energy harvester applications is described, found, and analyzed in these guidelines. The classification will give you a general idea of the research areas for piezoelectric energy harvesters. Furthermore, researchers should provide transparent views and indices for their research areas through the obstacles, limitations, and opportunities. In short, this study will be helpful for future research in the optimum design of piezoelectric energy harvesters depending on the application and the source of energy. It will also help to identify and overcome any existing limitations of such piezoelectric energy harvesters.

## Figures and Tables

**Table 1:** Comparison of different configurations for piezoelectric transducers [32].

Type of Configuration	Advantages	Disadvantages
Cantilever beam	Simple structure Low cost of fabrication Output power is proportional to proof mass Lower frequency of resonance The mechanical quality factor is high	Poor resistance to a high impact force
Circular diaphragm	Compatible with pressure mode operation	Stiffer compared to cantilever beam of the same size Higher frequencies of resonance
Cymbal type	High energy output Withstands high impact force	Limited to applications demanding high-magnitude vibration sources
Stacked type	Withstands high mechanical load Suitable for pressure mode operation Higher output from d_33_ mode	High stiffness

**Table 2:** Peak power generated by piezoelectric materials [32].

Piezoelectric Material	Peak Power (mW)	Volume	Frequency (Hz)	Reference
PVDF	0.61	72 × 16 × 0.41 mm	2	[72]
Piezoelectric ceramic	52	1.5 cm^3^	100	[73]
Piezoelectric fiber	120	2.2 cm^3^	-	[74]
PMN-PZT single crystal	0.015	20 × 5 × 0.5 mm	1744	[75]
PMN-PT single crystal	3.7	25 × 5 × 1 mm	102	[76]

**Table 3:** Different floor-based piezoelectric energy harvesters (PEH’s) [80].

Company-Technology	Product Dimension	Generated Power or Energy
Waynergy Floor	40 × 40 cm tile	10 W per step
Sustainable Energy Floor	50 × 50 cm tile	Typical power output for continuous stepping by a person lies between 1 and 10 W nominal output per module (average 7 W)
Pavegen tiles	V3 tile 50 cm each edge	5 Watts continuous power from footsteps
Electro-Active Polymers	Sheets	1 W
Sound Power	50 × 50 cm tile	0.1 W per 2 steps
PZT ceramic	Manufactured in small size	8.4 mW
Parquet PVDF layers	Layers	2.1 mWs per pulse with loads of about 70 kg
Drum Harvesters-Piezo buzzer, Piezoelectric ceramics	Vary	Around 2.465 mW
POWER leap PZT	Tile 24″ × 24″	0.5 mW per step

**Table 4:** Recent developments in PEH’s and their limitations.

Main Focus	Structure	Technique Adopted	Limitation	Reference
A strain-based energy harvester for the tire application inspired from Cymbal PEH.	Modified Cymbal structure with PZT-5H material.	A Multiphysics model was developed, with the FEM then validated with existing experimental results.	Depth of designed PEH cannot be increased to provide energy for more than two sensors due to the limitation of the maximum allowable size of the energy harvester connected to the tire’s inner surface.	[105]
Effect of position and dimension of piezoelectric buzzers embedded in shoe soles on energy harvesting.	PZT buzzer.	The Kistler force plate and the zebras’ force distribution measurement was utilized to calculate the force and pressure exerted by the foot on the sole via experimentation.	Electrical output is affected by the position and buzzer’s area inside the sole of the shoe.	[106]
A multimodal hybrid piezo-electromagnetic insole energy harvester to generate energy from the biomechanical energy wasted during daily walking.	Circular PZTs along with an upper and lower electromagnetic generator.	The structure was simulated for Eigen-frequency analysis using COMSO and the experimental setup was implemented to analyze the harvester’s performance at different resonant frequencies.	The harvester produces peak power at four resonant frequencies (8, 25, 50, and 51 Hz). The parameter tuning is necessary at higher resonant frequencies (25, 50, and 51 Hz).	[107]
The tile-based PEH’s function and the operational frequency of the PZT unimorph cantilever were converted.	PZT layer (40 × 20 × 0.3 mm^3^), 24 unimorph PZT piezoelectric cantilevers.	Energy harvesting floor using piezoelectric frequency up-converting mechanism test via experimentations.	Differences in the PZT cantilevers’ curvature and because of the large size of the energy harvesting floor, the overall energy output of PEH may be reduced.	[108]
Energy is extracted during two stages: when the foot is landing on the tile and moving up and away from the tile.	Piezoelectric bimorph cantilever (71 × 25.4 × 0.76 mm^3^)	Double-stage energy harvesting floor tile consisting of the cover plate, a movable part, which is set to move in the vertical direction via experimental test.	The amount of power generated is dependent on the weight of the pedestrian.	[109]
Novel piezoelectric material (PZN0.5C) designed to monitor the on/off feature of home appliances in real-time.	Unimorph cantilever type using optimized PZN0.5C thick films.	A novel material piezoelectric system was developed by a tape casting method.	The generated power is less than a milliwatt.	[110]
Flutter suppression is achieved primarily by piezoelectric actuation with energy harvesting.	NACA0014 type of aerofoil with an additional damping effect on the wing vibration.	Piezoelectric materials are embedded into wing structures for energy harvesting from flutter suppression and wing vibrations.	The energy output of PEH is dependent on the amplitude of the residual vibration after the active control of the wing.	[111]
Analysis of the energy harvested by the cantilevered flag and flutter instability subjected to the axial flow inside the wind tunnel.	Cantilevered flag with piezoelectric material.	Flag-flutter based piezoelectric (PZT) energy harvester.	For the designed flag, a hysteresis phenomenon has been observed. Also, the occurrence of flutter is sudden.	[112]
Energy generation from the piezoelectric seaweed driven by wave force of ocean or river	Piezoelectric seaweed made by piezoelectric polymer (PVDF)	The deformation of piezoelectric seaweed due to the wave force of the ocean or river generates electrical energy via ANSYS design model and meshing.	Energy generated by the piezoelectric seaweed varies with the direction of deformation, and both ends of the piezoelectric seaweed may be deformed in different directions.	[113]
Energy generation from offshore buoys using novel piezoelectric-based ocean wave energy harvesting.	Clamped-guided piezoelectric (PZT) beam structure.	The electromechanical equations of motion for the energy harvesting system are accurately derived from governing equations for ocean wave energy harvester and then validated by experimental results.	Tip-mass in the self-tuning buoy should be changed every month.	[114]
To produce energy from breaking ocean waves on a vertical face using a new low-volume piezoelectric beam-column energy harvester.	Clamped-guided piezoelectric (PZT) beam structure	A vibration-based FEM model for a piezoelectric harvesting system was proposed with two degrees of freedom and used to extract energy from the heave motion of breakwater.	Presented the proof of concept and detailed design of the system is yet to be investigated.	[115]
Piezoelectric wave-energy converter consisting of power generator, buoy, and frequency-up mechanism.	Clamped piezoelectric (PZT) film.	Conceptual design based on the component of the piezoelectric WEC and then experimental investigation.	PWEC generates electricity when the wave height is greater than 15 mm, and the frequency of the waves determines the output voltage duration.	[116]
Design and development of piezoelectric mechanical energy harvesters for use in implants.	Ribbons of piezoelectric material (PZT) printed on thin film.	Contraction and relaxation of the right ventricle resulting in the bending of PZT ribbons which generate electrical energy.	Special materials are required. The PZT material is brittle.	[117]
Evaluation of implantable PEH generating energy from the pulsation of ascending aorta through in vitro and in vivo studies.	Aluminium coated Flexible PVDF film.	Pulsating energy of the aorta deforms the PVDF film. The energy generated by the PEH depends upon the amount of deformation. The ascending aorta has maximal deformation amplitude in all the blood vessels and is chosen for the study.	The performance of the PEH depends on the tightness of the PEH wrapping the aorta.	[118,119]

## Data Availability

Not applicable.
